# A case of autoimmune lymphoproliferative syndrome in a 3 years-old patient

**DOI:** 10.1186/1546-0096-9-S1-P10

**Published:** 2011-09-14

**Authors:** IN Lavrentieva, SR Rodionovskaya, IN Tsymbal, MA Maschan, TA Visotskaya

**Affiliations:** 1 Children's Hospital №38 Federal Medical Biological Agency of Russia, Moscow, Russia; 2 Federal Research Center of Pediatric Hematology, Oncology and Immunology, Moscow, Russia

## Background

Autoimmune lymphoproliferative syndrome (ALPS) - a disease which is based on primary violations lymphocyte apoptosis caused by various molecular defects: mutation in the proapoptotic receptor Fas (la ALPS type) or its ligand - FasL (Ib type), a mutation in the gene caspase-10 (type II), or an unidentified defect in the signaling pathway of Fas-receptor (III type). The typical clinical picture: a non-cancerous chronic lymphadenopathy, splenomegaly, autoimmune defects, cytopenias, hypergammaglobulinemia with high serum IgA and increasing in the number of double negative TCR-alfa-betaCDZ + CD4-CD8-lymphocytes.

## Case report

We have observed a 3-years-old female patient suffering from autoimmune lymphoproliferative syndrome. Since the first year-old the child has had spontaneous episodes of febrile fever, last 2-3 days, progressive lymphadenopathy, hepatomegaly (+5,0 cm from the costal margin), splenomegaly (+5,0 cm from the costal margin). Laboratory tests showed anemia, leukopenia without altering leukocyte formula. Increase in LDH, serum ferritin, triglycerides, hypergammaglobulinemia. There was also a significant increase in IgA. The results of the bone marrow aspirate, bone biopsy, lymph node biopsy, immunohistochemical study of lymph nodes were normal. Autoimmunity markers were negative. As the results of laboratory tests was excluding viral infections (serology, PCR), Coombs-positive anemia, histiocytosis, lymphoma, storage diseases, the some of systemic autoinflammatory diseases (Hyper-IgD-syndrome, periodic fever).

Further studies were carried out for differential diagnosis of ALPS. In the study in the analysis of peripheral blood revealed an increased number of double negative TCR-alfa-betaCDZ + CD4-CD8-lymphocytes. Genetic testing was not detected homozygous mutations in the Fas receptor gene.

## Conclusions

Patients with accurate clinical picture of an autoimmune lymphoproliferative syndrome in the absence of homozygous mutations require careful monitoring of the dynamic, because a high risk of developing Hodgkin's disease and non-Hodgkin's lymphoma.

